# Composition and richness of the serum microbiome differ by age and link to systemic inflammation

**DOI:** 10.1007/s11357-018-0026-y

**Published:** 2018-06-05

**Authors:** Thomas W. Buford, Christy S. Carter, William J. VanDerPol, Dongquan Chen, Elliot J. Lefkowitz, Peter Eipers, Casey D. Morrow, Marcas M. Bamman

**Affiliations:** 1 Department of Medicine, University of Alabama at Birmingham, Birmingham, AL 35294 USA; 2 Biomedical Informatics, Center for Clinical and Translational Sciences, University of Alabama at Birmingham, Birmingham, AL USA; 3 Department of Microbiology, University of Alabama at Birmingham, Birmingham, AL USA; 4 Department of Cell, Developmental, and Integrative Biology, University of Alabama at Birmingham, Birmingham, AL USA

**Keywords:** Aging, Leaky gut, Microbiome, Microbiota, Inflammation

## Abstract

Advanced age has been associated with alterations to the microbiome within the intestinal tract as well as intestinal permeability (i.e., “leaky gut”). Prior studies suggest that intestinal permeability may contribute to increases in systemic inflammation—an aging hallmark—possibly via microorganisms entering the circulation. Yet, no studies exist describing the state of the circulating microbiome among older persons. To compare microbiota profiles in serum between healthy young (20–35 years, *n* = 24) and older adults (60–75 years, *n* = 24) as well as associations between differential microbial populations and prominent indices of age-related inflammation. Unweighted Unifrac analysis, a measure of β-diversity, revealed that microbial communities clustered differently between young and older adults. Several measures of α-diversity, including chao1 (*p* = 0.001), observed species (*p* = 0.001), and phylogenetic diversity (*p* = 0.002) differed between young and older adults. After correction for false discovery rate (FDR), age groups differed (all *p* values ≤ 0.016) in the relative abundance of the phyla *Bacteroidetes*, *SR1*, *Spirochaetes*, *Bacteria_Other*, *TM7*, and *Tenericutes*. Significant positive correlations (*p* values ≤ 0.017 after FDR correction) were observed between IGF1 and *Bacteroidetes* (ρ = 0.380), *Spirochaetes* (ρ = 0.528), SR1 (ρ = 0.410), and *TM7* (ρ = 0.399). Significant inverse correlations were observed for IL6 with *Bacteroidetes* (ρ = − 0.398) and *TM7* (ρ = − 0.423), as well as for TNFα with *Bacteroidetes* (ρ = − 0.344). Similar findings were observed at the class taxon. These data are the first to demonstrate that the richness and composition of the serum microbiome differ between young and older adults and that these factors are linked to indices of age-related inflammation.

## Introduction

Chronic low-grade inflammation is one of the most consistent biologic features of advanced age, evidenced by over 10,000 publications in this area (Buford 2017). Yet, despite the common recognition of the inflammatory phenomenon, the etiology of age-related inflammation remains poorly understood. Recently, a novel hypothesis has emerged from our group and others suggesting that increases in gut permeability (i.e., “leaky gut”) and subsequent release of intestinal contents into the circulation may be a primary contributor to increases in age-related inflammation (Buford 2017; Nicoletti 2015).

Aging is associated with several relevant changes to overall gut health including increases in intestinal permeability (Man et al. 2015; Nicoletti 2015) as well as changes to the stability of the gut microbiome (Biagi et al. 2010; Jeffery et al. 2016)—the aggregate genetic material of microorganisms residing within the intestinal tract which contribute to regulating host health (Human Microbiome Project Consortium 2012). These changes are relevant in the present context as recent evidence indicates that changes in microbial composition and density can alter immunity and inflammation distal to the intestine (Belkaid and Naik 2013). Indeed, early studies in humans reported cross-sectional associations between gut microbiome profiles and circulating inflammatory cytokines of older adults (Claesson et al. 2012; Rampelli et al. 2013). However, the mechanisms through which gut dysbiosis could contribute to chronic, low-grade inflammation were unclear.

Basic and pre-clinical studies have also suggested that intestinal permeability, coupled with altered microbiota profiles (Clark et al. 2015; Rera et al. 2012), may drive age-related increases in systemic inflammation. Very recently, Thevaranjan et al. (2017) published a seminal study in a mouse model definitively demonstrating that age-related gut microbial dysbiosis drives intestinal permeability, microbial translocation to the circulation, and ultimately systemic inflammation. Yet, despite these important pre-clinical studies, data are lacking to link intestinal permeability to inflammation in humans.

We recently published the first human evidence demonstrating that circulating concentrations of zonulin, a physiologic regulator of intestinal permeability, were higher—indicating greater permeability—among healthy older adults than younger peers (Qi et al. 2017). Furthermore, zonulin concentrations were positively associated with circulating concentrations of inflammatory cytokines tumor necrosis factor alpha (TNFα) and interleukin 6 (IL6) (Qi et al. 2017), two of the primary inflammatory cytokines consistently associated with the aging process. The objective of this study was to expand upon these findings by providing the first data comparing microbial DNA profiles within the circulation of healthy and older adults. We hypothesized that the microbiome found within serum would display age-related differences in measures of both alpha- and beta-diversity—key measures to detect differences in microbiomes between differing populations (Kumar et al. 2014). Moreover, we also aimed to identify specific microbial DNA abundances significantly associated with circulating concentrations of IL6 and TNFα as well as insulin-like growth factor 1 (IGF1)—a hormone known to be intricately related to inflammatory cytokine production (Maggio et al. 2013; Rajpathak et al. 2008) and recently reported to be stimulated by microbiota (Yan et al. 2016).

## Results

### Participant characteristics, diet, and inflammatory parameters

Data from a total of 48 participants was included in the study. Participant descriptive statistics are shown in Table 1. The young (*n* = 24) and older adult (*n* = 24) groups were balanced for sex. Participants in each group were of similar height and body mass, resulting in a similar mean body mass index between groups. Body fat percentage and fitness were significantly different (*p* < 0.05) between groups. Regarding dietary intake, a trend toward significance (*p* = 0.061) was observed for greater daily caloric intake among younger adults compared to older adults. Young adults consumed significantly more carbohydrate (mean difference: 43.6 kcal/day, *p* = 0.008), including significantly more fiber (mean difference: 4.2 g/day, *p* = 0.005) than older adults. No differences were observed in daily intake of fat or protein nor in specific sub-types of fats including cholesterol, saturated fat, or mono/polyunsaturated fats (data not shown). Serum concentrations of IL6, TNFα, and IGF1 are shown by group in Table 1. Significant group differences (*p* < 0.05) were observed for IL6 and IGF1, but not TNFα.

**Table 1:** Participant demographic characteristics and inflammatory parameters

	Young adults	Older adults
Age, years	27.8 ± 4.0	63.9 ± 3.2**
Female, *n*	14 (58.3%)	14 (58.3%)
Height, cm	170.2 ± 11.0	169.1 ± 10.9
Body mass, kg	72.7 ± 12.9	74.9 ± 15.0
Body mass index, kg/m^2^	25.0 ± 3.0	25.9 ± 3.2
Body fat, %	30.1 ± 10.4	35.9 ± 6.4*
VO_2_max, mL/O_2_/min	37.5 ± 8.4	27.0 ± 4.8**
Dietary intake		
Total intake, kcal/day	2003 ± 779	1767 ± 625
Carbohydrate, g/day	253.9 ± 103.6	210.2 ± 75.5*
Fiber, g/day	20.4 ± 8.3	16.2 ± 8.7
Fat, g/day	76.5 ± 41.2	67.8 ± 36.4
Protein, g/day	75.4 ± 33.9	73.1 ± 30.1*
Serum inflammatory parameters		
Interleukin 6, pg/mL	0.38 ± 0.19	0.52 ± 0.20*
Tumor necrosis factor α, pg/mL	2.02 ± 0.60	2.20 ± 0.44
Insulin-like growth factor 1, μg/L	365.4 ± 129.2	188.4 ± 82.3**

*VO2max* maximal respiratory capacity (i.e., fitness); *p < 0.05, **p < 0.0001

All values (mean ± SD)

### Microbial analyses—overall microbiome composition, β-diversity, and α-diversity

**Fig. 1:**
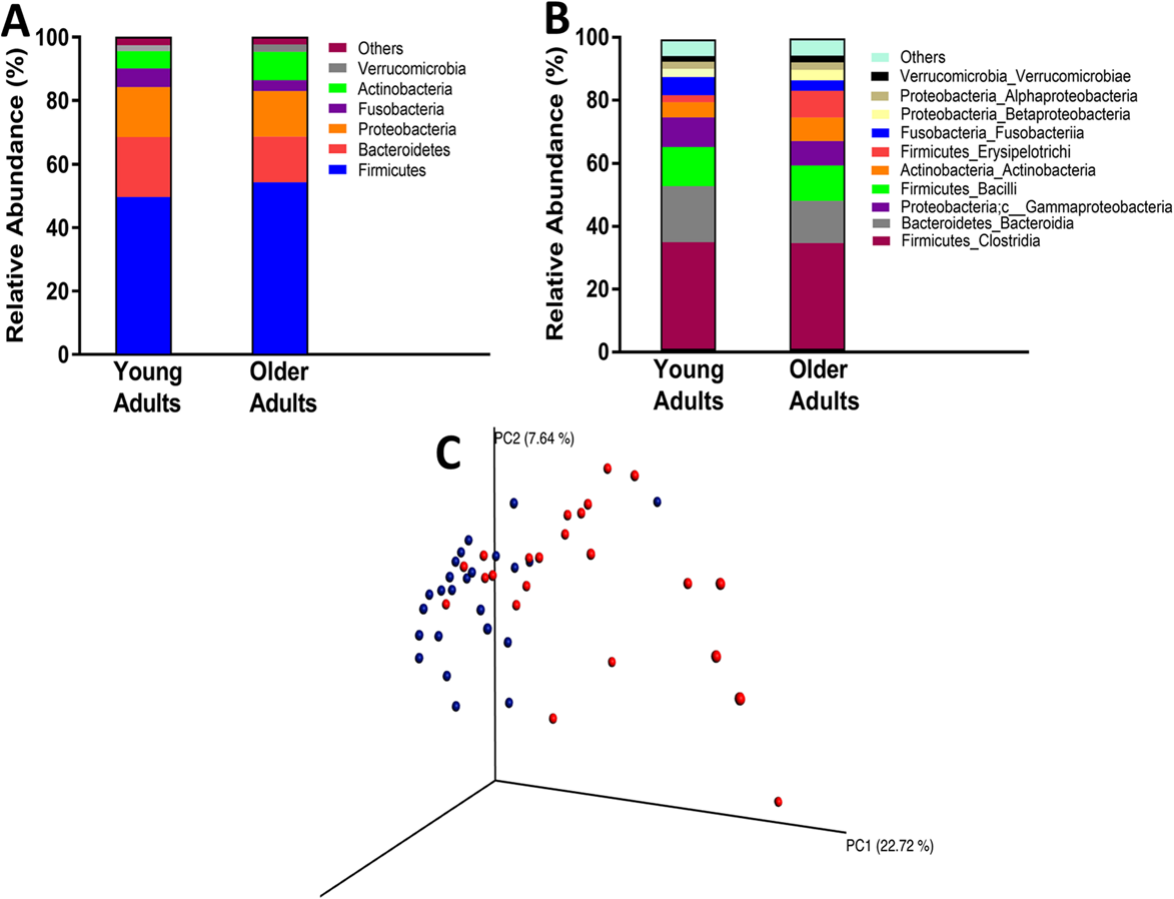
Taxonomic distribution of serum microbiome of healthy young and older adults by phylum (**a**) and class (**b**). **c** Comparison of serum microbiome β-diversity (Unweighted UniFrac) between healthy young (blue) and older (red) adults

**Fig. 2:**
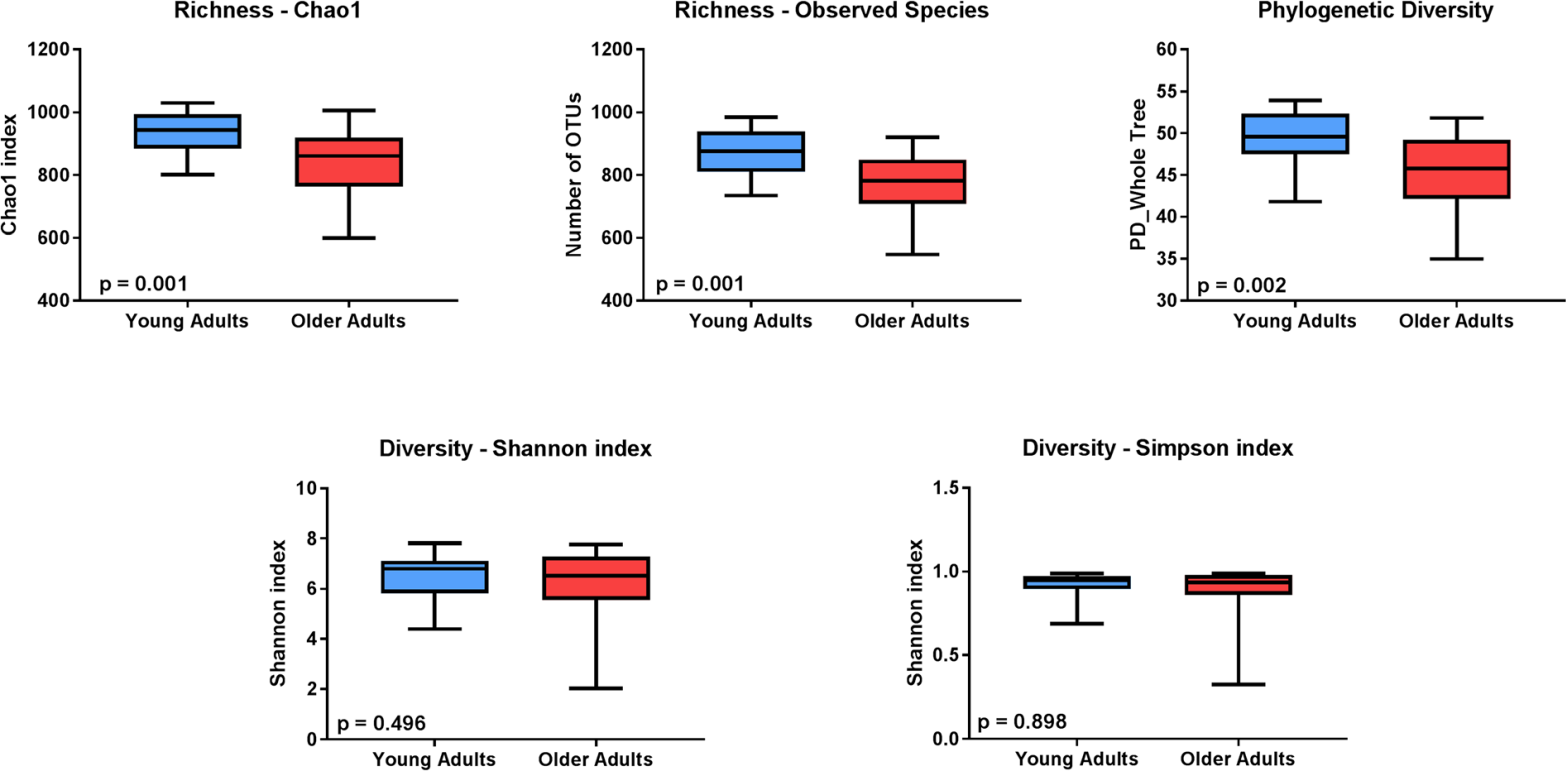
Comparison of α-diversity of the serum microbiome between healthy young (blue) and older adults (red). Five indices were used to represent the richness (chao1, observed species), phylogenetic diversity, and sample diversity (shannon and simpson indices). Box whiskers indicate the range of observed values

Figure 1 depicts the overall composition of the serum microbiomes among both young and older adults at both the phylum (A) and class (B) levels of taxonomy. Principal coordinate analysis (PCoA) revealed that age groups differed in the overall serum microbiota community structure as determined by *Unweighted UniFrac* (C). Key measures of α-diversity, including richness (chao1 and observed species) and phylogenetic diversity, were significantly different between young and older adults (Fig. 2). Overall sample diversity, measured according to the Shannon and Simpson metrics, did not significantly differ between age groups.

### Age-related differences in microbial abundances

**Fig. 3:**
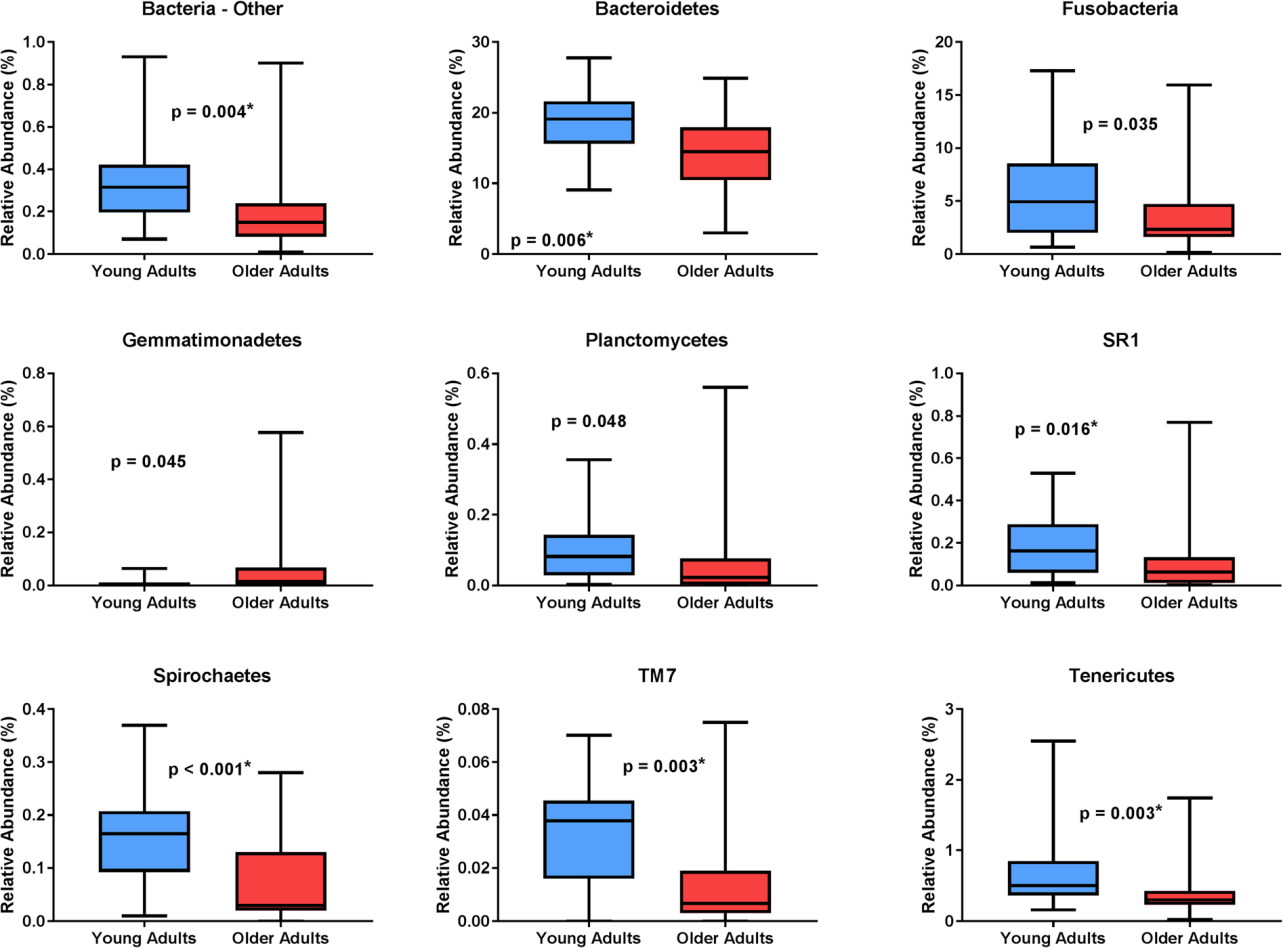
Microbial DNA populations differentially expressed between young (blue) and older (red) adults at the phylum level. Asterisk indicates statistical significance after correcting for multiple comparisons via false discovery rate. Box whiskers represent the range of observed values

The relative abundance of several bacterial phyla was significantly different between age groups (Fig. 3). After correction for false discovery rate (FDR), significant group differences were observed for the phyla *Bacteroidetes*, *SR1*, *Spirochaetes*, *Bacteria_Other*, *TM7*, and *Tenericutes.* At the class level, significant group differences were observed for *Bacteroidia*, *Mollicutes*, *Bacteria_Other_Other*, *Cytophagia*, *Firmicutes_Other*, and *Leptospirae* (Table 2)*.* Additionally, several other families with p values < 0.05 but not significant after FDR correction were identified, including *Erysipelotrichi*, *Fusobacteria*, *SR1_unknown*, and *Acidimicrobiia*.

**Table 2:** Serum microbiome composition at the class level (25 most common OTUs)

	Young adults	Older adults	*p* value for group
Firmicutes_Clostridia	34.8 ± 14.3	34.5 ± 15.4	0.932
Bacteroidetes_Bacteroidia	18.2 ± 4.7	13.6 ± 5.0	0.003*
Firmicutes_Bacilli	12.5 ± 7.0	11.4 ± 4.7	0.831
Proteobacteria_Gammaproteobacteria	9.6 ± 6.3	7.7 ± 3.2	0.580
Actinobacteria_Actinobacteria	4.9 ± 6.4	7.6 ± 7.4	0.093
Firmicutes_Erysipelotrichi	2.3 ± 1.6	8.6 ± 13.4	0.023
Fusobacteria_Fusobacteriia	5.9 ± 4.6	3.4 ± 3.6	0.035
Proteobacteria_Betaproteobacteria	2.7 ± 0.9	3.4 ± 2.3	0.496
Proteobacteria_Alphaproteobacteria	2.3 ± 2.3	2.4 ± 1.8	0.702
Verrucomicrobia_Verrucomicrobiae	1.9 ± 0.8	2.3 ± 1.3	0.217
Proteobacteria_Epsilonproteobacteria	0.8 ± 0.4	0.6 ± 0.4	0.085
Actinobacteria_Coriobacteriia	0.5 ± 0.2	0.6 ± 0.3	0.120
Bacteroidetes_Flavobacteriia	0.5 ± 0.2	0.6 ± 0.7	0.898
Cyanobacteria_Chloroplast	0.5 ± 0.4	0.7 ± 0.5	0.173
Tenericutes_Mollicutes	0.7 ± 0.5	0.5 ± 0.4	0.003*
Proteobacteria_Deltaproteobacteria	0.4 ± 0.3	0.4 ± 0.3	0.865
Bacteria_Other_Other	0.3 ± 0.2	0.2 ± 0.2	0.004*
Bacteria_SR1_unknown	0.2 ± 0.2	0.1 ± 0.2	0.016
Bacteroidetes_Cytophagia	0.04 ± 0.04	0.25 ± 0.49	0.003*
Cyanobacteria_Synechococcophycideae	0.07 ± 0.04	0.16 ± 0.22	0.328
Deferribacteres_Deferribacteres	0.10 ± 0.10	0.10 ± 0.08	0.686
Firmicutes_Other	0.11 ± 0.07	0.06 ± 0.05	0.011*
Spirochaetes_Leptospirae	0.10 ± 0.08	0.04 ± 0.06	0.001*
Actinobacteria_Acidimicrobiia	0.03 ± 0.03	0.04 ± 0.06	0.045
Cyanobacteria_Oscillatoriophycideae	0.02 ± 0.03	0.10 ± 0.20	0.034

All values (mean ± SD) indicate relative abundance (%)

*Statistically significant after correction for false discovery rate

### Associations of identified microbial communities with inflammatory parameters

**Fig. 4:**
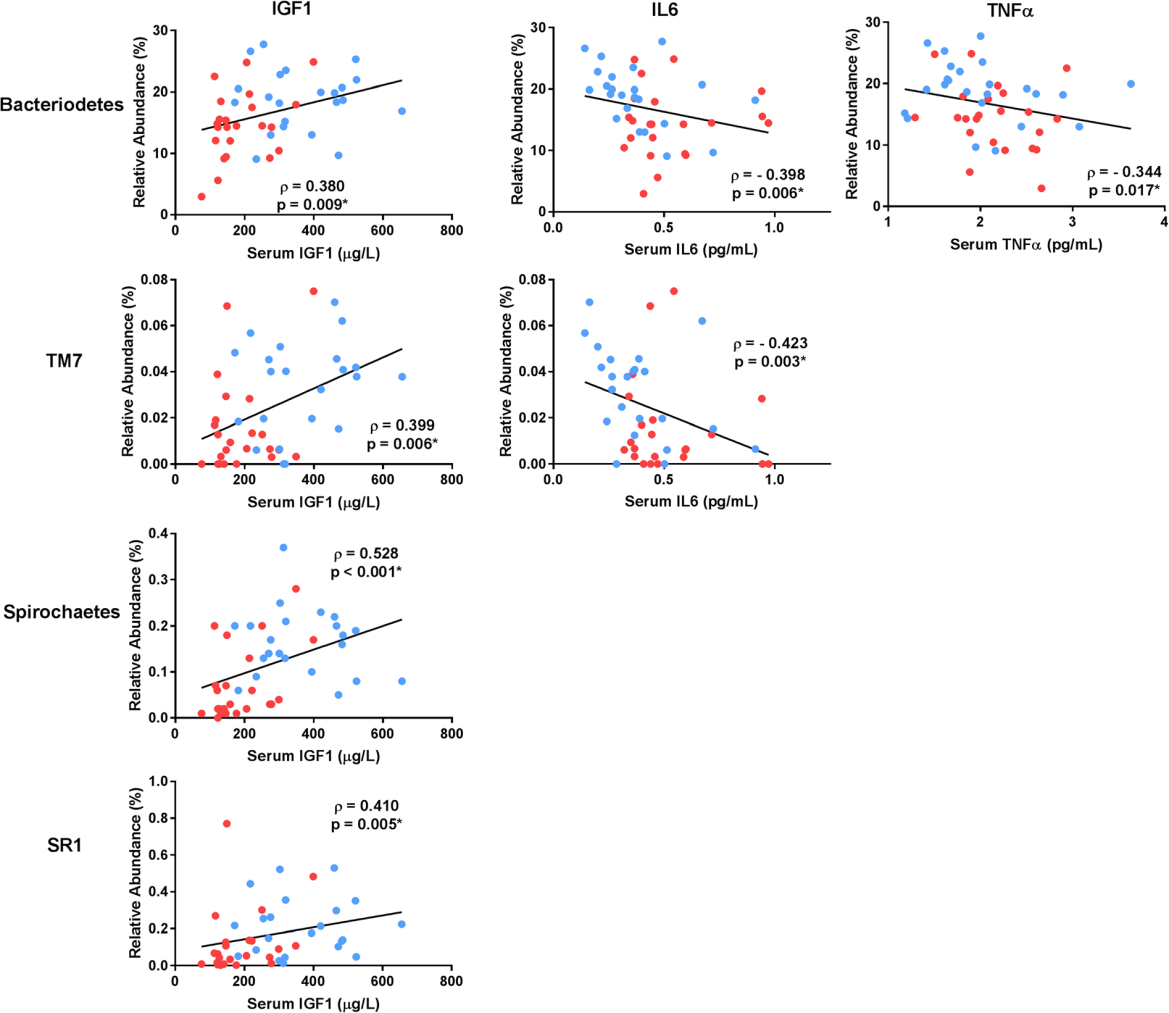
Microbial DNA populations at the phylum level significantly differing in abundance between young and older adults and correlated with indices of inflammation. Correlation coefficients reflect the Spearman rho comparison. Asterisk indicates statistical significance after correcting for multiple comparisons via false discovery rate. Data points are colored separately to indicate young (blue) and older (red) adults

**Fig. 5:**
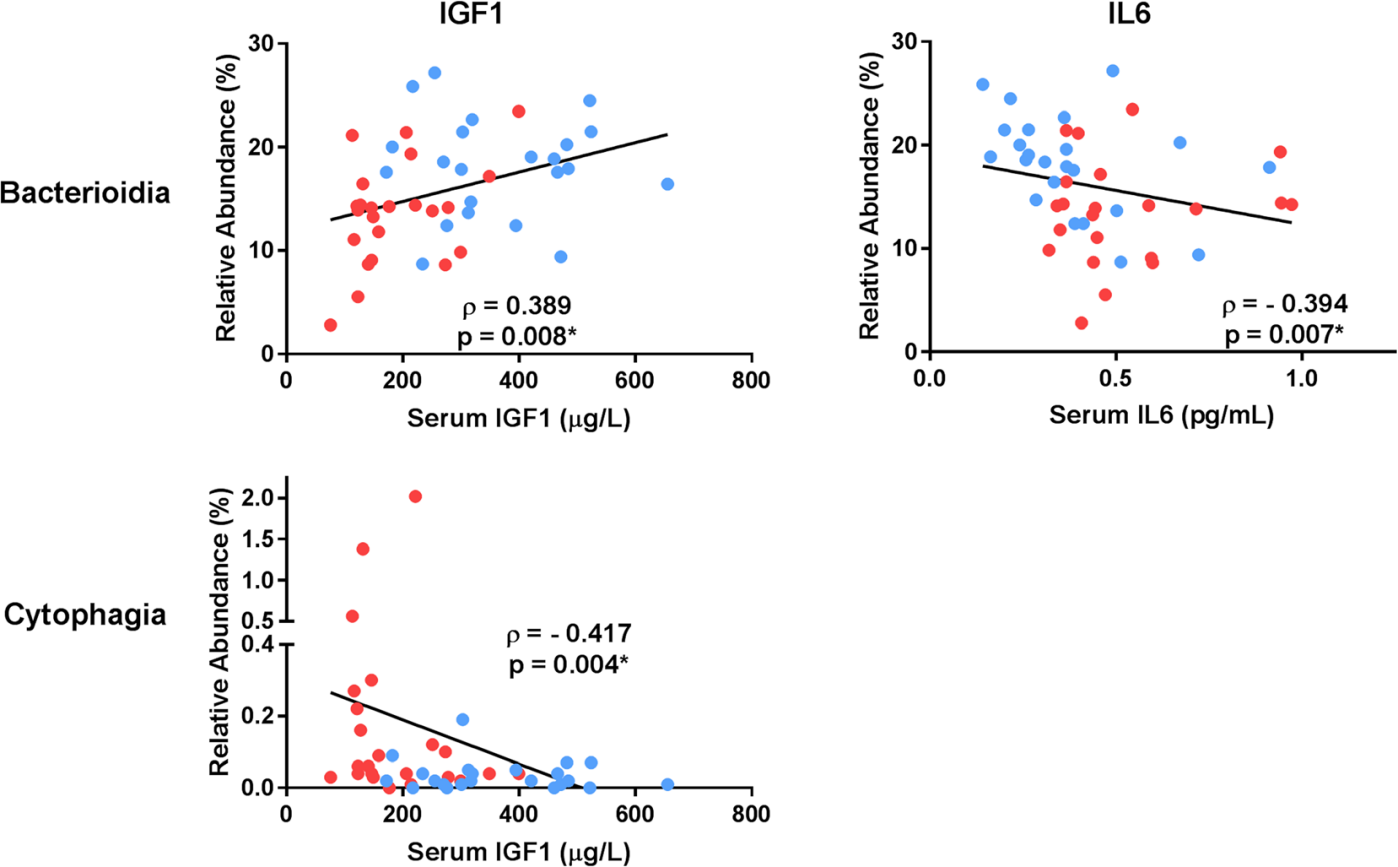
Microbial DNA populations at the class level significantly differing in abundance between young and older adults and correlated with indices of inflammation. Correlation coefficients reflect the Spearman rho comparison. Asterisk indicates statistical significance after correcting for multiple comparisons via false discovery rate. Data points are colored separately to indicate young (blue) and older (red) adults

Several phyla were significantly associated with serum inflammatory parameters (Fig. 4), in particular *Bacteriodetes* which was significantly correlated with all three measures. The phylum *TM7* was significantly correlated with both IGF1 and IL6. Additionally, several other phyla displayed *p* values < 0.05 but were not significant after FDR correction. These included the following: *Bacteria_Other* with IGF1 (ρ = 0.329, *p* = 0.025), *Tenericutes* with IGF1 (ρ = 0.303, *p* = 0.041), and *Spirochaetes* with TNFα (ρ = − 0.285, *p* = 0.050). At the class level, three significant correlations were observed including *Bacterioidia* with both IGF1 and IL6 as well as *Cytophagia* with IGF1 (Fig. 5). Correlations with other families with *p* values < 0.05 but not significant after FDR correction included *Bacteria_Other_Other* with IGF1 (ρ = 0.318, *p* = 0.033), *Leptospirae* with IGF1 (ρ = 0.321, *p* = 0.031), and *Bacterioidia* with TNFα (ρ = − 0.324, *p* = 0.026).

## Discussion

This is the first study to evaluate the age-related differences in microbial DNA profiles present in serum of healthy humans as well as associations of DNA abundances of specific microbial communities with indices of systemic inflammation. These findings are the first to indicate that the community structure of the microbiome in human serum differs between healthy young and older adults. Compared to younger adults, serum of older adults contained DNA from fewer species representing a lower level of phylogenetic diversity than that of young adults. Numerous bacterial phyla- and class-level differences were observed between age groups. Notably, the relative abundance of DNA from the *Bacteroidetes* phylum—one of the most abundant bacteria in both the gut and circulation—was significantly lower among older adults. Several of these differentially expressed bacterial DNA were also significantly correlated with indices of inflammation. DNA from *Bacteriodetes* in particular displayed strong relationships with inflammatory parameters as it was positively associated with IGF1 and negatively associated with both IL6 and TNFα.

Under healthy conditions, the compartmentalization of bacteria and other microbes to the gastrointestinal tract is maintained by a tight barrier at the intestinal-vascular interface (Spadoni et al. 2015). Yet, under certain clinical conditions, the integrity of this barrier can decrease and result in microbial translocation to the systemic circulation. For instance, microbial translocation due to a loss of immune control has been reported in HIV+ patients (Brenchley et al. 2006) as well as in cirrhotic patients with ascites (Santiago et al. 2016). In the case of the HIV+ population, microbial translocation was associated with low-grade systemic inflammation similar to findings observed in the in recent animal study of aging (Thevaranjan et al. 2017).

In the present study, the analysis performed from whole serum cannot differentiate between microbial DNA fragments and intact microbes. Even under healthy conditions, human blood contains bacterial DNA capable of triggering host innate immune responses (Hacker et al. 2002; Muruve et al. 2008; Nikkari et al. 2001). What is notable here, however, is the differences in the relative abundances between young and older adults. Several studies have reported alterations in circulating bacterial DNA abundances and corresponding immune/inflammatory profiles in patient populations including those with cirrhosis, kidney disease, and cardiovascular disease (Dinakaran et al. 2014; Frances et al. 2004; Kwan et al. 2013). In fact, differences in relative bacterial DNA abundances between patients and controls were proposed as an indicator of cirrhosis progression (Santiago et al. 2016). Though we cannot confirm the cause of these differentially expressed DNA, our prior findings related to zonulin concentrations in older adults as well as pre-clinical studies in this area lead us to hypothesize that these differences may be secondary to gut permeability. Future studies are needed to confirm this hypothesis.

Novel findings of this study include the differences in β-diversity as well as in the number of species with DNA expressed. In particular, DNA from the *Bacteroidetes* phylum differed by age and was significantly correlated with indices of inflammation. Given the lower abundance of *Bacteroidetes* DNA among older adults—these data could suggest a causal relationship between microbial DNA community composition and lower IGF1/higher inflammatory cytokines observed with advanced age. Though speculative, as a dominant microbial community, it is possible that reductions in circulating concentrations indicate increases in other potentially more reactive communities.

Another novel finding of the study is association of serum microbial DNA abundances with IGF1. Though typically known for its potent anabolic properties, IGF1 also has tremendous relevance to the human immune system. It is well documented that inflammatory cytokines attenuate IGF1 production (Maggio et al. 2013; Rajpathak et al. 2008), but IGF1 also plays an important role in regulating innate and acquired immunity—including the production of inflammatory cytokines (Heemskerk et al. 1999). Clinical data have recently implicated low IGF1 in flare-ups of inflammatory bowel disease (Krakowska-Stasiak et al. 2017), while basic studies have demonstrated that IGF1 directly inhibits pro-inflammatory cytokines in multiple animal cell types (Ji et al. 2017; Onnureddy et al. 2015), inducing LPS-induced cytokine expression (Onnureddy et al. 2015). This latter finding may have important implications for present findings, as microbial LPS may stimulate inflammatory cytokine production. Moreover, recent data reported that gut microbiota can stimulate IGF1 (Yan et al. 2016). Despite these links, the present data should not be over-interpreted as they do not provide any indication of directional causality. However, they do suggest that further follow-up may be warranted given the strength of associations and the aforementioned recent literature in this area.

Notably, dietary intake—including intake of dietary fiber—and fitness differed between young and older adults. Though these are differences commonly observed between young and older adults, these findings are important in the present context as diet and physical activity/exercise are among the primary factors known to influence gut microbiota communities (Campbell and Wisniewski 2017; Chen et al. 2014; O’Sullivan et al. 2015; Pallister and Spector 2016). To our knowledge, no data exist to directly indicate that diet or exercise can alter systemic bacterial DNA expression. However, both high-fat meals and highly vigorous exercise are known to be capable of inducing intestinal permeability, bacterial translocation, and even transient endotoxemia (Costa et al. 2017; Kelly et al. 2012). It is unclear at present how these factors might contribute to age-related differences in serum microbiome profiles, but these factors are important to consider for proper interpretation of study findings and in moving forward to causal studies.

As with any study, the present investigation is not without limitations. For instance, as noted above, the 16S microbiome analysis does not discriminate between microbial DNA fragment and intact microbes.

However, as noted, previous studies have shown that even bacterial DNA fragments are capable of stimulating immune reactions based on their foreign structure (Hacker et al. 2002; Muruve et al. 2008; Nikkari et al. 2001). Again, it is possible that differences in serum microbial DNA expression may be influenced by exercise (as evidenced by fitness) or diet which are important regulators of the intestinal microbiome. However, this is purely speculative at present. Additionally, only a single time-point was examined; thus, it remains unclear if serum microbe composition changes over time.

In summary, this study is the first to demonstrate age-related differences in the composition of the serum microbiome and associations between DNA expression of microbial communities and circulating indices of inflammation. Future studies are needed to evaluate causal links between these outcomes as well as associations between the abundance of microbial communities in the serum among those with various chronic diseases.

## Experimental procedures

### Study population

A total of 48 healthy, community-dwelling adults from the Birmingham, AL metropolitan area was included in this study. These participants represented a sub-set from a larger study protocol investigating skeletal muscle changes and exercise responsiveness with aging. Inclusion criteria were based on age ranges of 20–35 years for younger adults and 60–75 years for older adults. Subjects were free of chronic disease and not obese (body mass index < 30 kg/m^2^). All subjects completed health history questionnaires, and older adults passed a comprehensive physical exam and a diagnostic exercise stress test with 12-lead ECG to confirm health status. All participants were also assessed for body composition via dual x-ray absorptiometry and for aerobic fitness (i.e., VO_2_max) via a maximal exercise challenge with expired gases as further indicators of overall health status. Habitual dietary intake was assessed via 4-day food records analyzed using Nutrition Data Systems for Research (NDSR) software (Nutrition Coordinating Center, University of Minnesota, Minneapolis, MN). Prior to participation, all participants provided written informed based on documents approved by Institutional Review Boards of the University of Alabama at Birmingham (UAB) and Birmingham Veterans Affairs Medical Center.

### Blood collection and inflammatory analyses

Venous blood was collected and spun down to obtain serum using standard clinical practices. Serum IL6 and TNFα were determined using a Meso Scale Discovery (MSD; Rockville, MD) Quick Plex SQ 120 imager using electrochemiluminescence technology. Minimum sensitivity for the IL6 assay was 0.07 pg/mL, while sensitivity was 0.09 pg/mL for TNFα. Intra-assay coefficients of variation (CV) were 7.84 and 7.67%, and inter-assay coefficients were 5.78 and 2.5% for IL6 and TNFα, respectively. IGF1 was assessed via immunoradiometric assay (Diagnostic Systems Laboratories, Webster, TX). The inter-assay CV, intra-assay CV, and assay sensitivity for IGF1 were 9.43, 3.48, and 4.89 ng/mL, respectively.

### Microbiome analyses—16S PCR amplification

The 16S V4 analysis was done as previously described (Kumar et al. 2014). DNA was extracted from serum samples with the ZR Fecal DNA Miniprep Kit (Zymo Research, Irvine, CA) (Kumar et al. 2014). PCR was used with unique bar-coded primers to amplify the V4 region of the 16S rRNA gene to create an “amplicon library” from individual samples as described by Kumar et al. (2014). Cycling conditions for the PCR reactions were as follows: initial denature 94 °C for 1 min followed by 32 cycles of 94 °C for 30 s, 50 °C for 1 min, 65 °C for 1 min, and a final extension of 65 °C for 3 min. The entire PCR reaction was electrophoresed on a 1.0% agarose/Tris-borate-EDTA gel. The PCR product (approximately 250 base pairs) was visualized by UV illumination. The band was excised and purified from the agarose using Qiagen QIAquick Gel Extraction Kit according to the manufacturer’s instructions.

The PCR products were then sequenced using the Illumina MiSeq platform (Kumar et al. 2014). Paired end reads of approximately 250 bp from the V4 region of 16S rDNA were analyzed. The samples were first quantitated using Pico Green, adjusted to a concentration of 4 nM then used for sequencing on the Illumina MiSeq (Kumar et al. 2014). Fastq conversion of the raw data files was performed following de-multiplexing. Quality control of the fastq files was performed which was then subject to quality assessment and filtering using the FASTX toolkit (FASTX). The remainder of the steps was performed using the Quantitative Insight into Microbial Ecology (QIIME) suite, version 1.8 (Kumar et al. 2014; Lozupone et al. 2007; Navas-Molina et al. 2013). One sample was removed from analysis due to failing quality control procedures.

### Microbiome analyses—sequence data analysis and composition

The sequence data covered the 16S rRNA V4 region with a PCR product length of ~ 255 bases and 250 base paired-end reads. Since the overlap between fragments was approximately 245 bases, the information from both ends of the paired reads was merged to generate a single high-quality read using the module “fastq_mergepairs” of USEARCH (Edgar 2010). Read pairs with an overlap of less than 50 bases or with too many mismatches (> 20) in the overlapping region were discarded. Chimeric sequences were also filtered using the “identify_chimeric_seqs.py” module of USEARCH (Edgar 2010). Overall, read quality was assessed before and after filtering using FASTQC (*FASTQC.* *http://Www.bioinformatics.babraham.ac.uk/projects/fastqc/*)*. The QIIME* data analysis package was used for subsequent 16S rRNA data analysis (Caporaso et al. 2010a, b). Sequences were grouped into operational taxonomic units (OTUs) using the clustering program UCLUST at a similarity threshold of 0.97% (Edgar 2010). The Ribosomal Database Program (RDP) classifier was used to make taxonomic assignments (to the genus and/or species level) for all OTUs at confidence threshold of 80% (0.8) (Wang et al. 2007). The RDP classifier was trained using the Greengenes (v13_8) 16S rRNA database (McDonald et al. 2012).

The resulting OTU table included all OTUs, their taxonomic identification, and abundance information. OTUs whose average abundance was less than 0.0005% were filtered out. OTUs were then grouped together to summarize taxon abundance at different hierarchical levels of classification (e.g., phylum, class, etc). Multiple sequence alignment of OTUs was performed with PyNAST (Caporaso et al. 2010a, b). Alpha diversity (diversity within the samples) was calculated using Shannon’s diversity matrix which measures both richness (number of OTUs/species present in a sample) and evenness (relative abundance of different OTUs/species and their even distribution in a sample) (Jost 2007), as implemented in QIIME (Caporaso et al. 2010a, b). Beta diversity (diversity between the samples) was measured using unweighted Unifrac analysis (Lozupone and Knight 2005). Principal coordinate analysis (PCoA) was performed by QIIME to visualize the dissimilarity matrix between all samples, such that samples that were more similar were closer in space than samples that were more divergent. A 3D PCoA plot was generated using EMPEROR (Vazquez-Baeza et al. 2013).

### Statistical analysis

All data were evaluated for normality and homogeneity of variance prior to determination of descriptive statistics and comparative analyses. Group comparisons for demographic, dietary, and inflammatory data were performed using Student’s *t* tests for independent samples. The observed species metric of α-diversity was assessed using Student’s *t* test. Other indices of α-diversity were assessed via the Mann-Whitney test. A *p* value of < 0.05 was utilized to identify differences in descriptive data between groups. Comparison of microbial abundances between groups, both at the phylum and class levels, were analyzed using the non-parametric Mann-Whitney test. For the class level, only the 25 most common OTUs were evaluated due to the low abundance of other OTUs. A significance level of *p* < 0.05 was utilized for initial identification of OTUs of interest, with final determination of significance established after correcting for false discovery rate (FDR) according to the method of Benjamini and Hochberg (1995). Following comparative analyses, correlational analyses were performed among the inflammatory parameters and those OTUs identified (adjusted for FDR) as significantly differing in relative abundance between age groups. Correlation coefficients were calculated using the Spearman procedure. Correlations with *p* values < 0.05 were flagged, with final determination of significance established after correcting for FDR.
